# 2D BP/InSe Heterostructures as a Nonlinear Optical Material for Ultrafast Photonics

**DOI:** 10.3390/nano12111809

**Published:** 2022-05-25

**Authors:** Yiqing Shu, Zijun Zhong, Chunyang Ma, Penglai Guo, Leiming Wu, Zhitao Lin, Xun Yuan, Jianqing Li, Weicheng Chen, Quanlan Xiao

**Affiliations:** 1 School of Computer Science and Engineering, Macau University of Science and Technology, Avenida Wai Long, Taipa, Macao 591020, China; 1909853yii30001@student.must.edu.mo (Y.S.); penglai_guo@163.com (P.G.); 201004ly@gmail.com (Z.L.); xunyuan520butterflyjun@gmail.com (X.Y.); jqli@must.edu.mo (J.L.); 2 International Collaborative Laboratory of 2D Materials for Optoelectronics Science and Technology of Ministry of Education, Institute of Microscale Optoelectronics, Shenzhen University, Shenzhen 518060, China; 1809853gii30001@student.must.edu.mo; 3 Research Center of Circuits and Systems, Peng Cheng Laboratory (PCL), Shenzhen 518055, China; macy15@foxmail.com; 4 Institute of Advanced Photonics Technology, School of Information Engineering, Guangdong University of Technology, Guangzhou 510006, China; leiming_wu@gdut.edu.cn; 5 Zhuhai MUST Science & Technology Research Institute, Zhuhai 519000, China; 6 School of Physics and Optoelectronic Engineering, Foshan University, Foshan 528225, China; chenwch@fosu.edu.cn; 7 Guangdong-Hong Kong-Macao Joint Laboratory for Intelligent Micro-Nano Optoelectronic Technology, Foshan University, Foshan 528225, China

**Keywords:** BP, InSe, heterostructure, LPE, nonlinear optical responses, ultrafast photonics application, mode-locked pulse, dark-bright soliton pairs

## Abstract

The BP/InSe heterojunction has attracted the attention of many fields in successful combined high hole mobility of black phosphorus (BP) and high electron mobility of indium selenide (InSe), and enhanced the environmental stability of BP. Nevertheless, photonics research on the BP/InSe heterostructure was insufficient, while both components are considered promising in the field. In this work, a two-dimensional (2D) BP/InSe heterostructure was fabricated using the liquid-phase exfoliation method. Its linear and non-linear optical (NLO) absorption was characterized by ultraviolet−visible−infrared and Open-aperture Z-scan technology. On account of the revealed superior NLO properties, an SA based on 2D BP/InSe was prepared and embedded into an erbium-doped fiber laser, traditional soliton pulses were observed at 1.5 μm with the pulse duration of 881 fs. Furthermore, harmonic mode locking of bound solitons and dark-bright soliton pairs were also obtained in the same laser cavity due to the cross-coupling effect. The stable mode-locked operation can be maintained for several days, which overcome the low air stability of BP. This contribution further proves the excellent optical properties of 2D BP/InSe heterostructure and provides new probability of developing nano-photonics devices for the applications of double pulses laser source and long-distance information transmission.

## 1. Introduction

Black phosphorus (BP), a group-V mono-elemental material with puckered structure, possessing several advantages of adjustable bandgap, high carrier mobility as well as large on-off current ratios, has provided many applications in electronics, biomedicine, catalysis, optoelectronics, energy storage, sensors, etc. [1,2,3,4,5]. However, the inherent shortcoming of low chemical stability of BP leads to a major stumbling block for its applications in diverse environments, where it takes the risks of oxidation, photochemical reactions, and hydrolysis [6,7,8,9,10]. In order to overcome the shortcomings and further improve the performance of existing materials, heterostructures are pushed under the spotlight [11,12,13]. Superior to individual component materials, heterostructures maintain the intrinsic properties of each contained material due to in-plane strong covalent bonds and integrate the potential advantages by adjusting the out-of-plane stacking components held together by Van der Waals (vdW) forces [14,15,16]. Recently, mono- and few-layered indium selenide (InSe) with properties of small effective electron mass, and broad-band optical absorption have been successfully synthesized. Compared with BP, InSe processes higher electron carrier mobility up to ~10^3^ cm^2^ V^−1^ s^−1^. Constructing a BP/InSe heterostructure is a chance to not only combine the high electron mobility of InSe with the high hole mobility of BP, but also improve the long-term air stability of BP from days to many weeks [17]. Meanwhile, the band gap energy range of the direct (e.g., BP) and quasi-direct (e.g., InSe) two-dimensional (2D) layers is broadened markedly, which further indicates potentials in the field of ultra-broadband optoelectronics [17,18,19]. In 2017, Ding et al. demonstrated type-II band alignment and high carrier mobility of BP/InSe, thus predicted applications in field-effect transistors, photodetectors, and photovoltaic devices [19]. Today, thin-layered BP/InSe heterostructure has been practically used in diverse fields such as high polarization-sensitive photodetectors and giant quantum Hall effect devices [16,20,21]. In 2019, Cao et al. reported an optoelectronic device based on the BP/InSe heterostructure. Such a wide response range of 405–1550 nm, fast response speed of 22 ms and high response of 53.80 AW^−1^ at λ = 655 nm, and 43.11 AW^−1^ at λ = 1550 nm, excited optoelectronic device researchers [20]. However, there are few studies on BP/InSe heterostructures focused in the field of photonics. Of particular interest, the opportunity provided by this heterojunction as BP and InSe thin layers present a number of attractive features: size-dependent nonlinear saturable absorption and low saturation intensity are completely suited to exploit a novel optical device based on this material to generate ultrafast lasers [22,23,24,25,26].

Ultrafast lasers played a crucial role in various sophisticated technologies including ultra-precision manufacturing, ultrafine medical surgery, ultrafast information processing, and ultra-precision ranging [27,28,29,30,31]. Commonly used methods to generate ultrashort pulses are fiber lasers, solid-state lasers, quantum cascade lasers, optical parametric oscillators, and sum frequency generation [32,33,34,35,36,37,38,39,40,41,42]. Among them, fiber lasers have gained favor with researchers due to its unique advantages of light and flexible structure, high beam quality, and good heat exchange. Saturable absorbers (SAs) are one of the indispensable devices in fiber lasers to generate ultrashort pulses [43,44,45]. An ideal SA material should possess the following properties concurrently: good absorption properties, short recovery time, low mode-locking threshold, high damage threshold, a wide operating range of wavelengths, and low cost for mass preparation [46,47,48]. Specifically, the breakthroughs in the development of SA technology mainly owe to the improvement of SA materials. As mentioned above, the excellent optical properties of BP and InSe combined in the heterostructure hint at promising applications as SAs in generating ultrafast lasers [49,50].

In this research, a 2D layered Bp/InSe heterostructure material was fabricated using the liquid phase exfoliation (LPE) method [51,52]. Quality of the material was demonstrated via the characterization of transmission electron microscopy (TEM), Raman, and XPS. The stronger broadband linear and nonlinear-optical (NLO) absorptions of BP/InSe heterostructure were characterized by ultraviolet−visible−infrared (UV−vis−IR) and Open-aperture (OA) Z-scan technology. The superior nonlinear optical absorption of this heterostructure compared with most of previously reported 2D materials is underpinned by its large nonlinear absorption coefficient and low saturation intensity. Then, an Er-doped fiber ring cavity based on 2D Bp/InSe heterostructure was constructed and traditional soliton were obtained. Moreover, by changing the pump power and polarization states in the cavity, harmonic bound state pulses and dark bright soliton pairs were observed for the first time. These results affirm the excellent optical properties of BP/InSe heterostructure and enrich the diversity of SA family, and furthermore, provide more approaches for the research on nano-photonics devices of switches, detectors, photodiodes, and modulators.

## 2. Preparation and Characterization

**Figure 1:**
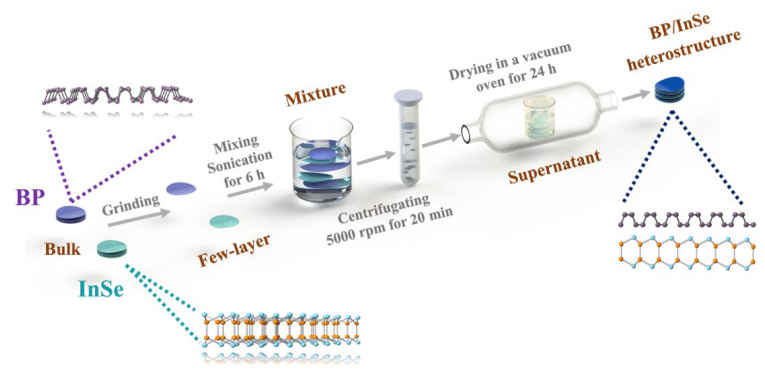
Schematic process for preparing 2D BP/InSe heterostructure by the LPE method.

2D BP/InSe heterostructure nanosheets were fabricated by the common method of LPE [53]. The preparation process is illustrated in Figure 1 in detail: bulk BP and InSe (BP/InSe~1:2) were ground into powders individually and mixed together. The mixture was sonicated with an ultrasound probe in isopropyl alcohol (IPA) for 6 h with the purpose of exfoliating 3D bulk particles into 2D layered nanosheets. Synchronously, the individual components of different materials were continually connecting by van der Waals force to forming heterostructures. The as-prepared suspension was centrifuged for 20 min at rotation speeds of 5000 rpm. Eventually, 2D BP/InSe heterostructure powder was obtained by drying the supernatant liquid in a vacuum oven at room temperature for 24 h.

**Figure 2:**
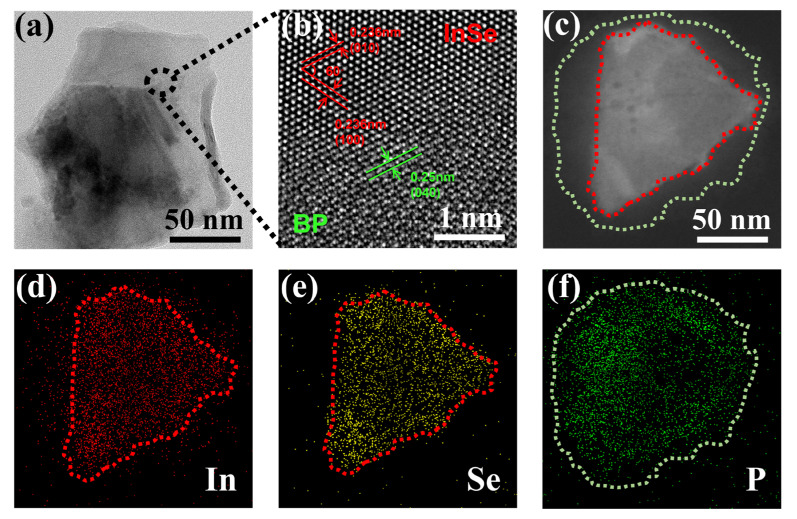
Morphology and characterization of the prepared 2D BP/InSe heterostructure. (**a**) TEM image. (**b**) HRTEM image. (**c**–**f**) Elemental mapping image.

The surface morphologies of the prepared 2D BP/InSe heterostructure were examined by transmission electron microscopy (TEM). Figure 2a is a typical TEM image of this material presenting the obvious layered structure in the span of 50 nm which means the bulk materials have been peeled off successfully. Figure 2b is the HRTEM image exhibiting the regular lattice structures of BP and InSe, the darker region illustrates the stacking of the BP and InSe nanosheets. The elemental mapping of In, Se, and P are shown in Figure 2c–f. The even distribution of the elements and the highly overlapped area identified the successful combination of BP and InSe.

**Figure 3:**
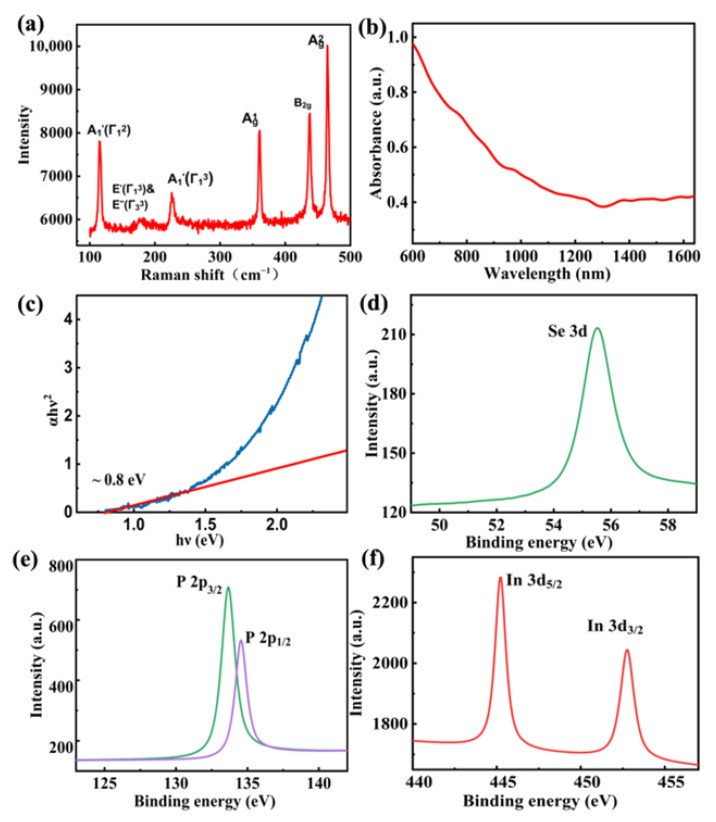
Characterization of the prepared 2D BP/InSe heterostructure. (**a**) Raman spectra. (**b**) UV-VIS-IR spectra. (**c**) Tauc plot of (αhν)^2^ versus hν. (**d**–**f**) XPS spectrum of In, Se, and P, respectively.

The identity of constructed BP/InSe heterostructure was also verified by Raman spectroscopy as seen from Figure 3a. Characteristic peaks of $A_{g}^{1}\;$(361 cm^−1^), $A_{g}^{2}$ (465 cm^−1^), $B_{2g}^{}$ (438 cm^−1^) correspond to BP, while ($A_{1}^{\prime }\left(\Gamma_{1}^{2}\right)$ (115 cm^−1^), $\;E^{\prime }\left(\Gamma_{1}^{3}\right)$ &$\;E\prime \prime \left(\Gamma_{3}^{3}\right)\;$(176 cm^−1^)_,_ ${\;A}_{1}^{\prime }\left(\Gamma_{1}^{3}\right)\;$(226 cm^−1^)) correspond to InSe. These results are consistent with the findings of previous works and further suggest that BP is free from oxidation in this sample [54,55]. The broadband optical absorption of the BP/InSe heterostructure was carried out by a UV–VIS–IR spectrometer from 600 to 1650 nm shown in Figure 3b. The optical bandgap was calculated to be ~0.8 eV (corresponding to wavelengths of ~1550 nm) with the Tauc method depicted in Figure 3c [53], which indicates that the optical response band of 2D BP/InSe heterostructure can be consistent with the working band of Er-doped fiber laser. Figure 3b–d show the XPS spectrums of this heterostructure. The binding energies peaks of 445.22 eV, 452.76 eV, 133.55 eV, 134.6 eV, and 55.52 eV are contributed by In 3d_5/2_, In 3d_3/2_, P 2p_3/2_, P 2p_1/2_, and Se 3d, respectively. Relative to the XPS peaks of individual BP and InSe in previous works, the peaks of constructed BP/InSe heterostructure changed visibly owing to electron transfer between layers of different components, which further confirms a successful synthesis of the BP/InSe heterojunction [56,57,58].

## 3. Nonlinear Optical Responses

**Figure 4:**
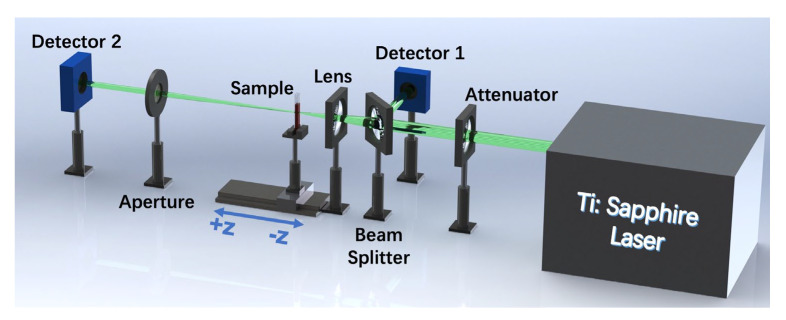
Schematic diagram of the OA Z-scan.

To verify the NLO response of the 2D BP/InSe heterostructure, a series of OA Z-scan techniques was used for characterizing [59,60,61]. The experimental setup is shown in Figure 4. The whole signal was measured by detectors and the Z-dependent signal variation was entirely contributed by the nonlinear absorption of the sample.

**Figure 5:**
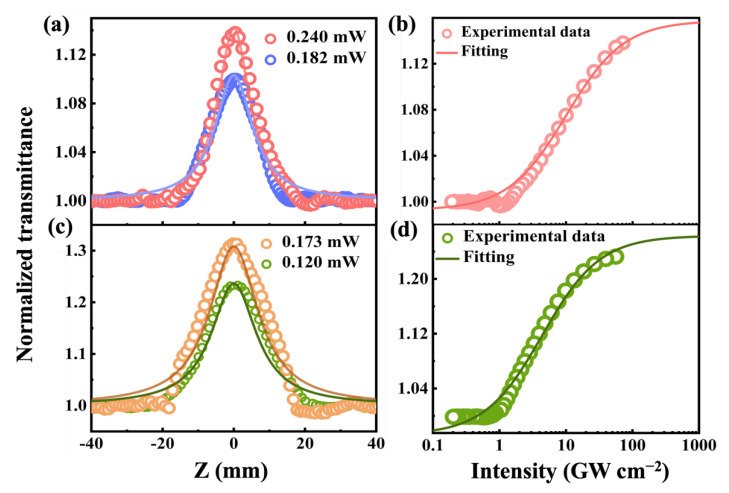
OA Z-scan measurements of 2D BP/InSe heterostructure at (**a**) 800 and (**c**) 1500 nm. Relationship between normalized transmittance of 2D BP/InSe heterostructure and input peak intensity of the femtosecond laser at (**b**) 800 and (**d**) 1500 nm.

Experimental data of the OA Z-scan measurement at 800 and 1550 nm are exhibited in Figure 5. Obviously, the normalized transmittance gradually increases with the increasing distances between the focus (*z* = 0) and the sample, showing typical optical saturable absorption features. The numerical values of nonlinear absorption coefficient (β) were obtained by fitting the experimental data with this following formula [62]:

(1) $$T\left(z\right)=1-\beta I_{0}L_{eff}/\left[2^{\frac{3}{2}}\left(1+z^{2}/z_{0}^{2}\right)\right]\times \pi \times w_{0}^{2}\times 10^{-10}$$

where T(z) is the normalized transmittance, $I_{0}$ is the peak on-axis power at z = 0 and $z_{0}$ is the Rayleigh range. $L_{eff}$ and $w_{0}$ are effective length and waist radius, respectively. The values of β were calculated greater than 10^−2^ cm/GW at the wavelength from 800 nm to 1550 nm. This order of magnitude is comparable to other benchmark NLO materials of BP, MoS_2_, graphene, MOFs. For further appraising the applicability of the sample as a potential SA, many required parameters as modulation depth (*T_s_*), saturation intensity (*I_s_*), and nonsaturable loss (*T_ns_*) are evaluated according to the single-photon absorption model:

(2) $$T=1-T_{s}\left(1+I/I_{S}\right)-T_{ns}$$

where T is the transmittance and I is the incident laser intensity. The function relationship between T and I is directly presented in Figure 5, and the data are exhibited in Table 1. Comparing these values of β, $I_{S}$ with original BP and other 2D materials, BP/InSe heterostructure possesses advantages of high β and low $I_{S}$, as seen in Table 2, indicating that BP/InSe heterostructure is competent to be a SA.

**Table 1:** NLO parameters of BP/InSe at different wavelengths.

λ (nm)	β (cm/GW)	*Is* (GW/cm^2^)	$T_{s}$ (%)	$T_{ns}$ (%)
800	−0.11	9.86	16.82	15.74
1550	−1.3 × 10^−2^	10.07	38.04	24.74

**Table 2:** Summary of *β* and *I_s_* values for various different NLO materials.

	λ (nm)	β (cm/GW)	*Is* (GW/cm^2^)	References
BP	800	−(0.68 ± 0.02) × 10^−3^	334.6 ± 43	[44]
MoS_2_	800	−(4.6 ± 0.27) × 10^−3^	413 ± 24	[63]
MOFs	800	−3 × 10^−2^	30	[64]
Ge	800	−(1.53 ± 0.31) × 10^−4^	16.4 ± 0.2	[65]
Bi_2_Te_3_/FeTe_2_	800	−7.53 × 10^−4^	314	[66]
BP/Ti_3_C_2_	800	-0.675	30.1	[67]
BP/InSe	800	−0.11	9.86	This work

## 4. Ultrafast Photonics Application in Fiber Lasers

**Figure 6:**
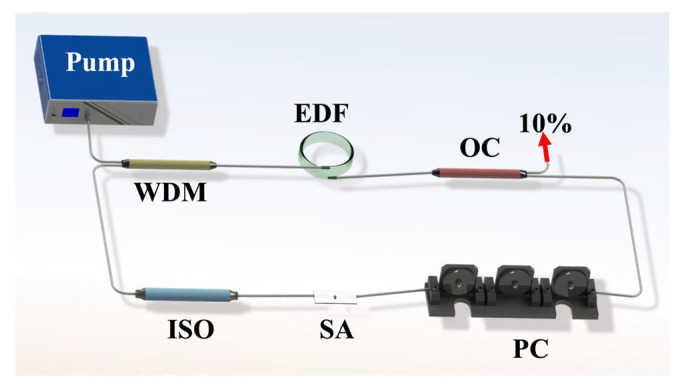
The schematic illustration of the EDF ring cavity.

Based on the outstanding NLO characteristics of the 2D BP/InSe heterostructure with low *I_s_* and large *T_s_*, a tapered fiber coated with this material was prepared and integrated into an erbium-doped fiber (EDF) ring cavity as an SA. The schematic illustration of the cavity is shown in Figure 6. Various kinds of ultrashort pulses were generated and their performances were evaluated.

A 980-nm laser source was selected to pump a 0.4-m-long gain fiber of EDF through a wavelength division multiplexer (WDM), then followed by a 10/90 fiber optical coupler (OC) to output the generated pulses. The Bp/InSe SA was embedded in the cavity between a polarization controller (PC) and a polarization independent isolator (ISO), which were utilized to control the cavity birefringence and ensure the unidirectional operation of the ring cavity, respectively. By changing the pump power and adjusting the polarization state, a variety of stable solitons was obtained in the EDF laser.

### 4.1. Typical Mode-Locked Pulse and 11th Harmonic Mode Locking of Bound State

**Figure 7:**
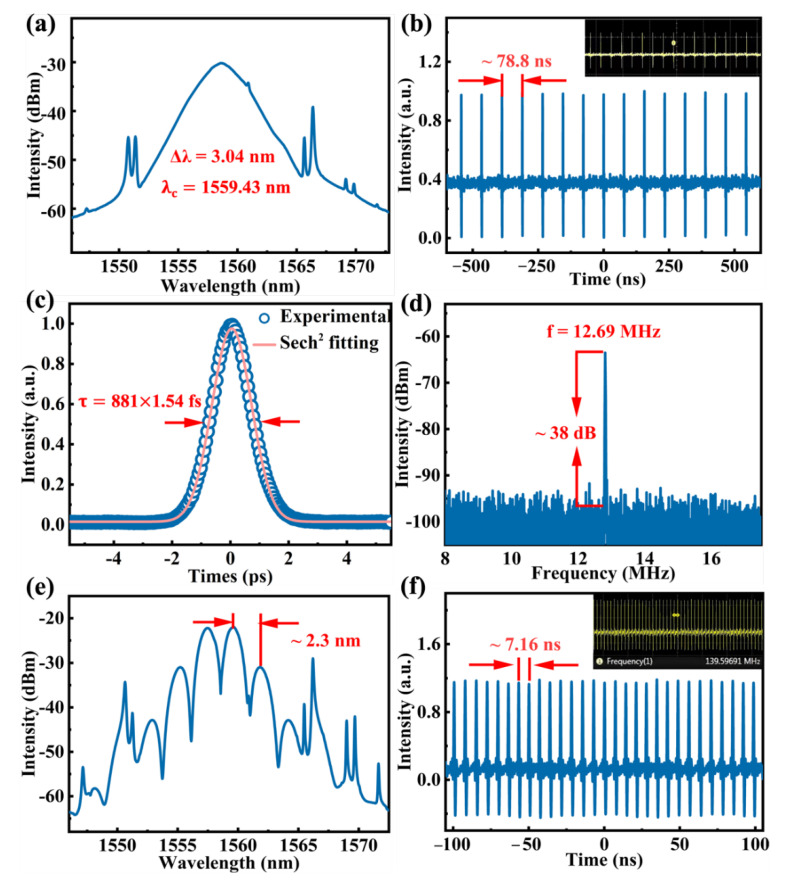
Typical mode-locked pulse output characteristics of the EDF laser based on 2D Bp/InSe SA. (**a**) Optical spectrum. (**b**) Pulse train. (**c**) Autocorrelation trace. (**d**) Radiofrequency (RF) spectrum. The 11th harmonic mode locking of bound state output characteristics of the EDF laser based on 2D Bp/InSe SA. (**e**) Optical spectrum. (**f**) Pulse train.

When the pump power was 240 mw and the corresponding output power was 4.6 mW, a traditional soliton with center wavelength of 1559.43 nm was generated. The measured spectrum with symmetric pairs of Kelly sidebands is depicted in Figure 7a, and its corresponding 3 dB spectral width is 3.04 nm. The corresponding mode-locked pulse sequence, in a span of 1250 ns and pulse interval of ~78.8 ns coinciding with the cavity length of 16.3 m, was measured by a real-time oscilloscope, as shown in Figure 7b. The pulse duration was obtained using a commercial autocorrelator. The experimental data were fitted with the Sech^2^ formula and the pulse duration was estimated to be 881 fs, illustrated in Figure 7c. The corresponding time-bandwidth product (TBP) of the soliton pulse can be calculated by the following equation [43]

TBP = *τ_pulse_* × *c* • Δ*λ*/*λ_c_*^2^ (3)

where *c*, Δ*λ*, and *λ_c_* represent the light speed, 3 dB bandwidth, and center wavelength of the optical spectrum. These parameters in this experiment are *τ_pulse_* = 881 fs, Δ*λ* = 3.04 nm, Δ*λ* = 1559.43 nm, respectively. The TBP is calculated to be ~0.33 (>0.315), indicating a weak chirp. The radio frequency (RF) spectrum of the output pulse was measured by a spectrum analyzer to investigate the operation stability of the soliton pulse. The signal-to-noise ratio (SNR) was about 38 dB and the fundamental peak was located at the repetition rate of 12.69 MHz, as shown in Figure 7d.

Under the circumstance of consistent pump power, by changing the polarization state in the cavity by slightly altering the orientation of PC, harmonic mode locking (HML) of bound solitons (BSs) was observed [68,69]. As shown in Figure 7e,f, the spectrum modulation period is 2.3 nm, the pulse interval is about 7.16 ns corresponding to the repetition rate of 139.6 MHz, which is about 11 times the fundamental repetition rate of the traditional soliton mode-locking. Different from the traditional soliton of single pulse, solitons in the state of HML uniformly distribute and repel each other in a long distance when evolving in the laser cavity. In the state of BSs, multiple solitons are bound to form one unit as a bound state soliton, and every unit propagates in the cavity with the same speed and discrete intervals [70]. Significantly, HML of BSs, different from the single-pulse HML or BSs, possess advantages of ultra-short separation and tunable intervals between two pulses. It is beneficial to obtain a double pulses laser source and push the boundaries of applications possibility in many fields [70].

### 4.2. Dark–Bright Soliton Pairs

**Figure 8:**
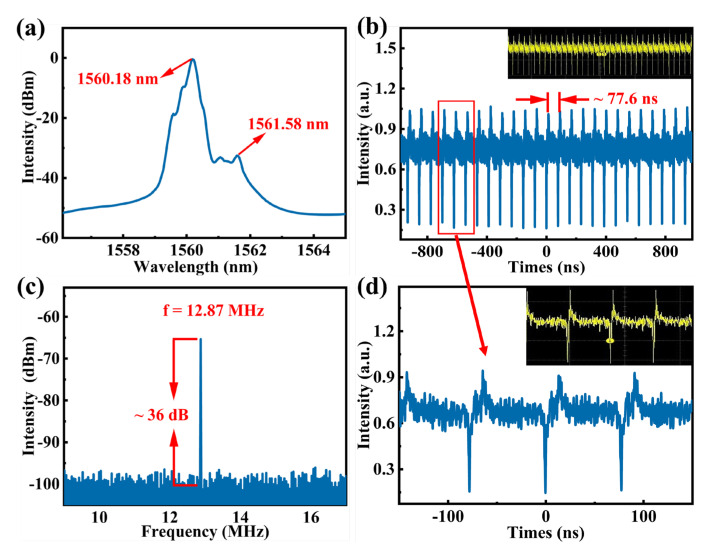
Typical mode-locked pulse output characteristics of the EDF laser based on 2D Bp/InSe SA. (**a**) Optical spectrum. (**b**) Pulse train. (**c**) Radiofrequency (RF) spectrum. (**d**) The pulse train with the span of 300 ns.

Compared with the generating process of bright solitons (a mode locked pulse is required in prior), dark solitons can be formed more easily, caused by a weak intensity dip of environment noise or the mode beating in the ring fiber laser [71]. Dark solitons possess charming advantages such as good stability under perturbations and less sensitivity to the background. Bright and dark solitons can be coupled into dark–bright soliton pairs due to the cross-coupling effect [72]. By increasing the pump power to 380 mW, with corresponding output power being 8.23 mW, dark–bright soliton pairs were generated. The pulse spectrum shown in Figure 8a contains two wavebands which is the combined result of the birefringence, filtering effect, and laser gain in the fiber cavity. The center wavelengths are located at 1560.18 nm and 1561.58 nm without obvious Kelly sidebands, corresponding to the bright and dark solitons, respectively [72]. This is consistent with the description of bright and dark soliton pairs spectrum reported previously [73,74]. The typical pulse train presented in Figure 8b with the pulse interval of ~78.8 ns coincides perfectly with the fundamental frequency of 12 MHz measured in the frequency domain shown in Figure 8c, and the SNR of the pulse is about 36 dB. Complementarily, the enlarged view of pulse pairs is displayed in Figure 8d to further characterize the dark–bright soliton pairs. Nevertheless, the pulse width of dark–bright soliton pairs was not captured by the conventional autocorrelation technique. This was possibly the result of the light–dark pulse pair’s inherent characteristics in fiber lasers, as mentioned in the previous reports [72,75]. The generation of dark–bright soliton pairs suggests that the nano-photonics devices based on 2D BP/InSe heterostructure hold auspicious potentials in long-distance information transmission as carriers [76]. More remarkably, the laser could keep operating in a stable state a week later, indicating a high damage threshold of the SA and a high oxidation resistance of 2D BP/InSe heterostructure.

## 5. Conclusions

In summary, a high-quality 2D BP/InSe heterostructure was prepared successfully by the LPE method and the superior NLO characteristics of 2D BP/InSe heterostructure were researched using the OA Z-scan technique. Its NLO characteristics of large *β* and low *I_s_* indicate that 2D BP/InSe heterostructure is provided with greater potential than benchmark optical materials to be an ideal SA. By integrating the SA into an EDF laser as a mode-locker, traditional soliton pulses were observed at 1.5 μm with the pulse duration of 881 fs. Furthermore, harmonic mode locking of bound solitons and dark–bright soliton pairs were obtained in the same laser cavity for the first time. The stable operability, lasting for several days, of this fiber laser demonstrates a higher antioxidant property of 2D BP/InSe than that of BP. Moreover, the rich soliton pulse behaviors not only confirm the excellent NLO characteristics of 2D BP/InSe heterostructure but is also beneficial to the research and development of double pulses laser sources and long-distance information transmission. In addition, it provides a meaningful reference for further improving the performance of the laser cavity including shorting the pulse width, increasing the repetition frequency, and increasing the peak power.

## Data Availability

The data presented in this study are available on request from the corresponding author.
